# Clinical characteristics of four cancer patients with SARS-CoV-2 infection in Wuhan, China

**DOI:** 10.1186/s40249-020-00707-1

**Published:** 2020-07-02

**Authors:** Shi-Hui Song, Tie-Long Chen, Li-Ping Deng, Yong-Xi Zhang, Ping-Zheng Mo, Shi-Cheng Gao, Wen-Jia Hu, Yong Xiong, Zhi-Yong Ma

**Affiliations:** Department of Infectious Diseases, Zhongnan Hospital of Wuhan University, Donghu road 169, Wuhan, 430071 China

**Keywords:** Severe acute respiratory syndrome coronavirus 2, Coronavirus disease 2019, Cancer, Wuhan

## Abstract

**Background:**

The severe acute respiratory syndrome coronavirus 2 (SARS-CoV-2) led to the outbreak of pneumonia in Wuhan. The virus is highly infectious. Patients with cancer might be susceptible to the viral infection because of the immunosuppressive state cause by therapies on tumors.

**Case presentation:**

We present the clinical features of four cancer patients who were infected with SARS-CoV-2 in late January of 2020 in our hospital. Cases 1 and 3 were diagnosed as mild and common type of coronavirus disease 2019 (COVID-2019) and survived from the viral infection. They acquired SARS-CoV-2 infection during their staying in hospital under radiotherapy and surgery of the tumors. Cases 2 and 4 suffered from severe type of COVID-19, and Case 2 was dead owning to the advanced age, uncontrolled chronic B cell lymphocytic leukemia and many other underlying diseases. The immunosuppressive state induced by liver transplantation and anti-rejection therapy might contribute to the severity of COVID-19 in Case 4, who suffered from hepatitis B related hepatocellular carcinoma. However, Case 4 was recovered from COVID-19 after a combination therapy against virus, bacteria and fungi, and also respiratory support. Nearly all patients showed a decrease in lymphocytes including total CD3^+^ T cells, B cells, and natural killer cells after infection of the virus.

**Conclusions:**

The severity of COVID-19 might be influenced by immune system state and underlying diseases in cancer patients. And the treatment of SARS-CoV-2 infection in cancer patients is challenged by the immunosuppressive state of these patients under chemotherapy or surgery.

## Background

In December 2019, a cluster of cases of pneumonia was reported by Wuhan Municipal Health Commission, China, and then a novel coronavirus was eventually identified [1, 2]. The severe acute respiratory syndrome coronavirus 2 (SARS-CoV-2) is highly infectious and can infect all individuals. Patients infected with the virus have suffered from the coronavirus disease 2019 (COVID-19), and have had symptoms including fever, cough, shortness of breath, diarrhea and vomiting [3]. As of 19 February 2020, there were 75 204 cases globally in 25 countries and the virus has led to 2009 deaths due to its rapid spread [4].

The development of COVID-19 has caused more severe cases and deaths in older people with underlying diseases, including diabetes, cardiovascular disease, and cancer [3]. There is currently no definite effective drug against the virus, although some drugs such as remdesivir, chloroquine phosphate, arbidol, lopinavir and ritonavir have been used in clinical practice and have exerted antiviral effects in some patients [5]. However, the antiviral efficacy of these drugs needs to be verified by large-scale studies. Recent studies have demonstrated a higher frequency of severe cases and increased mortality in patients in Wuhan compared with other regions of China [6].

Patients with cancer are thought to be more susceptible to infection than the general population because of their system’s immunosuppressive state cause by chemotherapy, radiotherapy, or surgery on tumors [7]. Here, we report on four cancer patients infected with SARS-CoV-2 in late January of 2020 and describe their medical history, clinical diagnosis, changes in clinical parameters, and outcomes.

## Case presentation

### Case 1

**Fig. 1:**
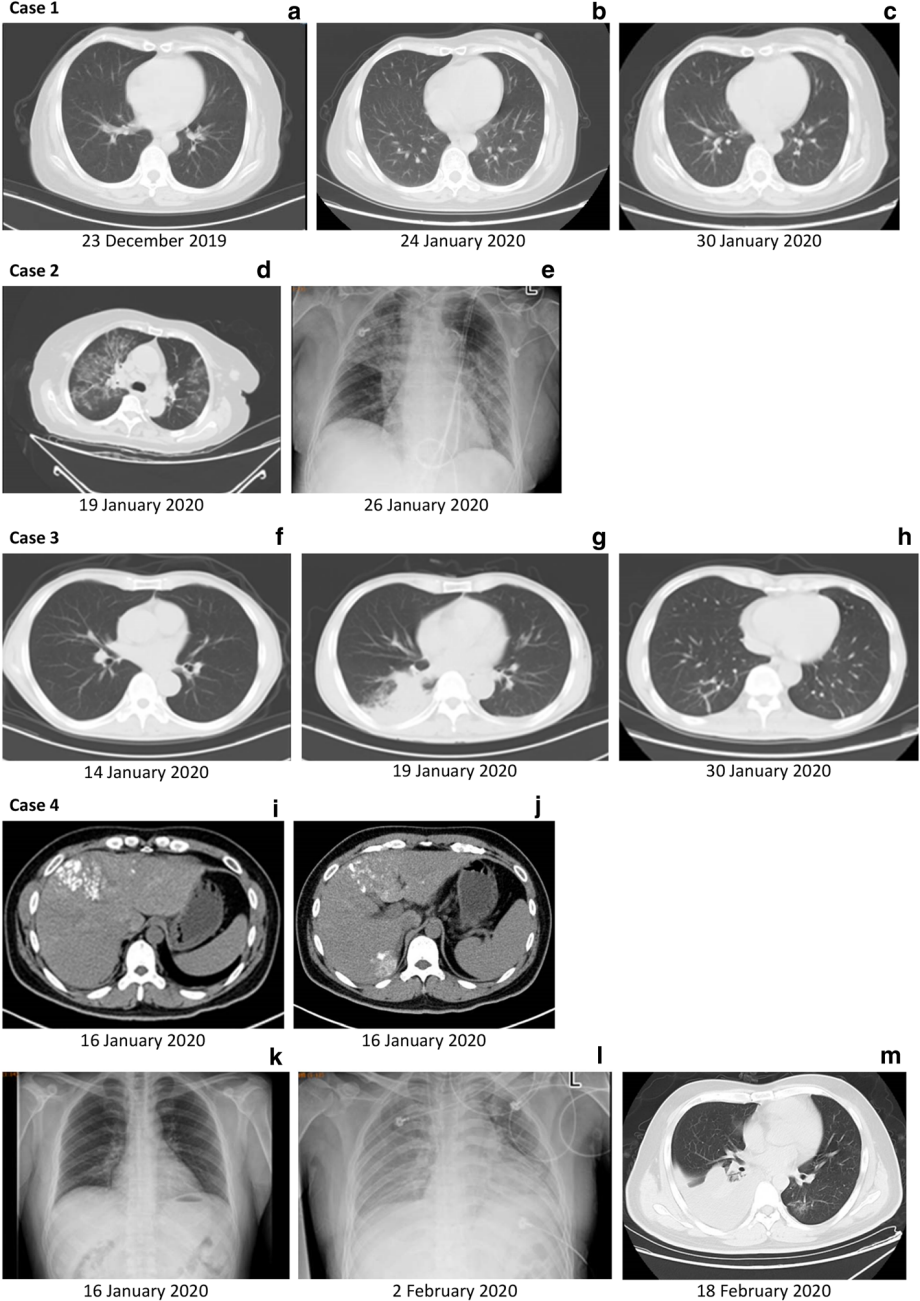
The chest X-ray/computed tomographic (CT) images of the four patients on different time points. **a**–**c** Images from Case 1. The patient had normal CT images on 23 December 2019 after admission (**a**). However, on 24 January 2020, after onset of fever, CT scan showed interstitial abnormalities in both lower lung, which revealed the possibility of viral pneumonia (**b**). And these changes were ameliorated after treatment and showed on 30 January 2020 (**c**). **d**–**e** Images from Case 2. The CT images showed bilateral patchy shadowing in the patient after admission (**d**), and the lesion progressed 1 week later (**e**). **f**–**h** Images from Case 3. The CT scans taken on different time points, showed local patchy shadowing in right lung 6 days after admission (**g**), which is the time point of fever onset in this patient. And the lesion was recovered after treatment and showed on 30 January 2020 (H). **i**–**m** Images from Case 4. **i**–**j** The CT scans showed the multiple HCC lesions in the liver after transcatheter arterial chemoembolization therapy. The X-ray examination of the patient revealed normal lung after admission (**k**). However, on 2 February 2020 the images from X-ray showed bilateral patchy shadowing, indicating viral pneumonia (**l**). The patient has been in remission of the pneumonia despite right pleural effusion was showed by CT scan on 18 February 2020 (**m**)

A 48-year-old woman was admitted to the Department of Radiation and Medical Oncology in our hospital on 12 December 2019, because of the need for continued radiotherapy for breast cancer. She received a modified radical mastectomy of the right breast on 25 June 2019, as well as six cycles of chemotherapy with paclitaxel and doxorubicin after surgery. After 4 weeks of radiotherapy, the patient presented fever on 24 January 2020, and this was accompanied by cough. Chest computed tomographic (CT) scans found slight interstitial abnormalities in both lower lungs, indicating a possibility of viral pneumonia (Fig. 1a, b). The SARS-CoV-2 infection was confirmed by positive detection of the virus in throat swab sample using the real-time reverse transcription polymerase chain reaction (RT-PCR) method. The laboratory results revealed reductions in white blood cells (WBCs) and lymphocytes in the blood, especially CD3^+^CD8^+^ T cells, B cells, and natural killer (NK) cells (Table 2). The patient was then diagnosed as suffering from COVID-19 and transferred to an isolation ward in the Department of Infectious Diseases. The symptoms were relieved after 2 days. The antibiotic ceftriaxone was given as empirical antibacterial therapy and ceased after relief of symptoms (Table 1). The chest CT scan on 30 January 2020 also suggested recovery from COVID-19 (Fig. 1c). However, the virus was sustained in the throat swab samples for several days (Table 2). The patient was transferred to another isolation ward, which was arranged by the government after discharge from our hospital. At last, the SARS-CoV-2 was cleared from the patient on 13 February 2020 (Table 2).

**Table 1:** Clinical characteristics of the four cancer patients with SARS-CoV-2 infection

Clinical characteristics	Case 1	Case 2	Case 3	Case 4
Sex	Female	Female	Male	Male
Age (years)	48	78	54	37
Date of admission	21 December 2019	18 January 2020	13 January 2020	14 January 2020
Date of COVID-19 diagnosis	26 January 2020	25 January 2020	23 January 2020	1 February 2020
Underlying diseases				
Hypertension	No	Yes	No	No
Cardiovascular disease	No	Yes	No	No
COPD	No	Yes	No	No
HBV infection	No	No	Yes	Yes
Tumor type	Breast cancer	B-CLL	Rectal cancer	HCC
Tumor related therapy				
Chemotherapy	Yes	No	No	Yes
Radiotherapy	Yes	No	No	No
Surgery	Yes	No	Yes	Yes
Date of surgery	25 June 2019	/	16 January 2020	20 January 2020
Dates of fever (day after admission)	Day 35, Day 36	Day 6–Day 17	Day 7–Day 11	Day 17–Day 24
Maximum temperature	38 °C	39 °C	39.5 °C	39 °C
Clinical type of COVID-19	Mild	Severe	Common	Severe
Anti-microbe therapy				
Antiviral	No	Oseltamivir	Oseltamivir	Oseltamivir Arbidol
Antibacterial	Ceftriaxone	Cefoperazone and sulbactam Linezolid	Meropenem Moxifloxacin	Imipenem and cilastatin Moxifloxacin
Antifungal	No	Caspofungin	No	Caspofungin
Methylprednisolone	No	40 mg/day	No	40 mg/day
Oxygen therapy	No	Noninvasive ventilation	Nasal catheter	High-flow oxygen
Minimal oxygenation index (mmHg)	NA	112	NA	261
ICU admission	No	Yes	No	Yes
Clinical outcomes	Recovery of COVID-19; Discharge from hospital	Dead	Recovery of COVID-19; Discharge from hospital	Remission of COVID-19; Therapy in hospital

*Abbreviations: SARS-CoV-2* severe acute respiratory syndrome coronavirus 2, *COVID-19* coronavirus disease 2019, *COPD* chronic obstructive pulmonary disease, *HBV* hepatitis B virus, *B-CLL* chronic B cell lymphocytic leukemia, *HCC* hepatocellular carcinoma, *ICU* intensive care unit, *NA* not available

**Table 2:** Laboratory and radiographic findings of the four cancer patients with SARS-CoV-2 infection

Radiographic and laboratory findings	Normal Range	Case 1	Case 2	Case 3	Case 4
**After admission**					
White blood cell count, × 10^9^/L	3.5–9.5	4.21	10.91↑	4.86	5.46
Neutrophil count, × 10^9^/L	1.8–6.3	2.05	4.11	3.8	3.53
Lymphocyte count, × 10^9^/L	1.1–3.2	1.3	5.85	0.57↓	1.2
Hemoglobin, g/L	130–175	101↓	118↓	133	152
Platelet count, ×10^9^/L	125–350	350	202	180	182
Procalcitonin, ng/mL	< 0.05	< 0.05	0.1↑	< 0.05	0.25
C-reactive protein, mg/L	0–10	NA	36.2↑	NA	NA
Abnormalities on chest X-ray/CT	No	No (Fig. 1a)	Bilateral patchy shadowing (Fig. 1d)	No (Fig. 1f)	No (Fig. 1k)
**After fever onset**					
White blood cell count, ×10^9^/L	3.5–9.5	2.86↓	6.67	2.7↓	9.36
Neutrophil count, ×10^9^/L	1.8–6.3	1.79↓	3.55	1.21↓	8.73
Lymphocyte count, ×10^9^/L	1.1–3.2	0.4↓	2.4	0.88↓	0.28↓
Hemoglobin, g/L	130–175	114↓	112↓	115↓	102↓
Platelet count, ×10^9^/L	125–350	156	177	280	50↓
Procalcitonin, ng/mL	< 0.05	< 0.05	0.57↑	< 0.05	< 0.05
C-reactive protein, mg/L	0–10	0.7	77.9↑	1.3	18.1↑
Influenza A or B virus detection	Negative	Negative	Negative	Negative	Influenza A virus (+)
1–3-β-D polyglucosan (pg/ml)	(Neg.) < 60 (Pos.) > 110	NA	< 10	NA	165.7↑
Abnormalities on chest X-ray/CT	No	Interstitial abnormalities (Fig. 1b)	Progress of bilateral lung diseases (Fig. 1e)	Local patchy shadowing (Fig. 1g)	Bilateral patchy shadowing (Fig. 1l)
Lymphocyte subsets (count/μl)					
CD3^+^T cells	805–4459	435↓	297↓	865	335↓
CD3^+^CD4^+^T cells	345–2350	290↓	115↓	482	148↓
CD3^+^CD8^+^T cells	345–2350	142↓	184↓	341↓	185↓
B cells	240–1317	12↓	4213↑	44↓	41↓
NK cells	210–1514	59↓	132↓	154↓	43↓
**Before discharge from hospital or dead**					
White blood cell count, ×10^9^/L	3.5–9.5	3.12↓	16.52↑	4.04	6.28
Neutrophil count, ×10^9^/L	1.8–6.3	2.18	7.37↑	2.22	5.34
Lymphocyte count, ×10^9^/L	1.1–3.2	0.41↓	7.94↑	1.07↓	0.55↓
Hemoglobin, g/L	130–175	110.1↓	98.7↓	107.3↓	133.5
Platelet count, ×10^9^/L	125–350	156	44↓	151	92↓
Procalcitonin, ng/ml	< 0.05	< 0.05	1.48↑	< 0.05	NA
C-reactive protein, mg/L	0–10	0.7	138.4↑	NA	NA
**Detection of SARS-CoV-2 in throat swab samples**					
Dates of positive results	/	26 January 30 January 3 February 8 February	25 January 28 January 1 February 5 February	23 January 30 January 3 February 9 February	1 February 4 February 8 February 12 February
Negativity time of SARS-CoV-2^a^	/	13 February	/	15 February	10 March

*Abbreviations: SARS-CoV-2* severe acute respiratory syndrome coronavirus 2, *NA* not available, *CT* computed tomographic, *NK cells* natural killer cells

^a^The negativity time of SARS-CoV-2 was defined as the first day of a negative test if the nucleic acid of SARS-CoV-2 was negative for 2 consecutive tests (the sampling interval is at least 1 day)

### Case 2

A 78-year-old woman from Wuhan came to our hospital with fatigue, malaise, and poor appetite on 18 January 2020. She was admitted to the Department of Hematology because of her past medical history of chronic B cell lymphocytic leukemia (B-CLL) for 5 years. She was also suffering hypertension, cardiovascular disease, and chronic obstructive pulmonary disease (COPD) for more than 10 years. She received percutaneous coronary intervention on 23 February 2014. She took aspirin and atorvastatin orally every day, and took nifedipine sustained-release tablets and indapamide sustained-release tablets to control blood pressure. Because of the presentation of the critical underlying diseases, she did not receive any treatment for B-CLL. The chest CT images found bilateral patchy shadowing, indicating double pneumonia in this patient on 19 January 2020 (Fig. 1d). The patient was diagnosed as suffering from double pneumonia, COPD, hypertension, B-CLL, and coronary heart disease, and received antibiotic therapy with oseltamivir, cefoperazone, sulbactam, linezolid, and caspofungin. Because of the immunocompromised state and the severe pulmonary infection in this patient, the initial empirical antimicrobial treatment covered influenza virus, bacteria and also fungi. However, the pneumonia progressed, and symptoms such as fever, shortness of breath, and dyspnea appeared in the patient on 25 January 2020 (Fig. 1e). The SARS-CoV-2 was found in a throat swab sample by real-time PCR. The patient was then transferred to an intensive care unit (ICU), and high flow humidification oxygen inhalation therapy and methylprednisolone (40 mg daily) were employed as combination therapy. After 5 days of therapy in the ICU, symptoms of dyspnea and hypoxia improved, and the patient was transferred to an isolation ward in our department on 31 January 2020. We continued the combination therapy employed in the ICU. However, the patient died on 10 February 2020 due to respiratory failure, despite the noninvasive ventilation (Table 1). The lymphocyte subset analysis in this patient showed an increase in B cells, which may indicate uncontrolled B-CLL (Table 2). The older age, multiple underlying diseases, and severe pulmonary infection might have contributed to the death of the patient.

### Case 3

A 54-year-old man from Wuhan was admitted to the Department of Colorectal and Anal Surgery on 13 January 2020. Six days before admission, a large polyp was found in his rectum by colonoscopy examination. He has been a hepatitis B surface antigen carrier for several years. He received a laparoscopic radical resection of rectal cancer on 16 January 2020, and the pathological result diagnosed rectal adenocarcinoma. On 19 January 2020, the patient presented fever, with no other symptoms. The fever might be caused by postoperative abdominal infection in this patient, so he received meropenem and moxifloxacin as antibacterial therapy. The chest CT scans suggested local patchy shadowing in the double lower lung, which might be the result of bacterial or viral infection (Fig. 1g). The oseltamivir was prescribed to the patient against the influenza virus. However, the SARS-CoV-2 was found in a throat swab sample by real-time PCR on 23 January 2020. The patient was then diagnosed as having COVID-19 and transferred to an isolation ward of our department. The patient also received oxygen therapy by nasal catheter in the isolation ward (Table 1). The fever stopped after 7 days of therapy, and the recovery of COVID-19 was confirmed by a chest CT scan on 30 January 2020 (Fig. 1h). The laboratory results also suggested a decrease in peripheral blood lymphocytes, such as B cells and NK cells, after SARS-CoV-2 infection (Table 2). The positive detection of SARS-CoV-2 in the throat swab samples of this patient lasted at least 18 days (Table 2). The patient was transferred to another isolation ward, which was arranged by the government after discharge from our hospital. The patient cleared the virus from throat swab sample 23 days after the first time of positive detection of SARS-CoV-2 (Table 2).

### Case 4

A 37-year-old man from Wuhan had the chief complaint of upper abdominal intermittent pain for more than 3 months. One day before admission to the Department of Hepatobiliary Surgery, a space-occupying lesion was found in the liver of this patient by ultrasound. He has been a hepatitis B virus carrier for more than 19 years and does not take antiviral drugs or see a doctor regularly. After admission, he was diagnosed as suffering from chronic hepatitis B and hepatocellular carcinoma (HCC). On 16 January 2020, the patient received chemotherapy through transcatheter arterial chemoembolization. Multiple hepatic HCC lesions were found in this patient through a CT scan afterward (Fig. 1i-j). Positron emission computerized tomography and computer tomography was performed, and no HCC lesion was found outside the liver. The patient received an allogeneic liver transplantation on 20 January 2020 for a better prognosis. A combination therapy with antibiotics, antiviral treatment with entecavir, a high dose of hepatitis B immunoglobulin, and immunosuppressive agent tacrolimus were given to the patient after surgery. However, the patient began to present a fever on 30 January 2020, and this was accompanied by a cough. The nucleic acids of influenza A virus and SARS-CoV-2 were detected in throat swab samples on 31 January and 1 Febuary 2020. The patient was transferred to an ICU on 2 February 2020 due to dyspnea, and the chest X-ray showed bilateral patchy shadowing, indicating COVID-19 (Fig. 1l).

In the ICU, the patient was treated with a combination therapy including high-flow humidification oxygen inhalation, antibiotic therapy with oseltamivir, arbidol, imipenem, cilastatin, moxifloxacin, caspofungin, and methylprednisolone (40 mg daily) (Table 1). Because the patient had increased plasma level of 1–3-β-D polyglucosan, and positive detection of influenza A virus and SARS-CoV-2 (Table 2), he received a combination antimicrobial therapy. Two days later, the symptoms were relieved in this patient. The patient was transferred to an isolation ward in our department on 4 Febuary 2020, and received the same treatment employed in the ICU. On 19 Febuary 2020, we ceased all antibiotic therapy and methylprednisolone. The lung CT images showed remission of the pneumonia despite the right pleural effusion found on 18 Febuary 2020 (Fig. 1m). Consistent with laboratory findings of other patients, the lymphocytes including T cells, B cells, and NK cells decreased after infection with SARS-CoV-2 in this patient (Table 2). The virus was persistent in the samples from throat swabs in this patient, so the patient remained in the hospital (Table 2). Finally, the patient cleared SARS-CoV-2 in throat swab samples on 10 March 2020, about 39 days after the first time of positive detection of the virus (Table 2).

## Discussion and conclusions

In the present study, we collected the clinical data from four cancer patients who were infected by SARS-CoV-2 and developed COVID-19. The diagnosis of COVID-19 was based on symptoms, lung X-ray/CT examination, and detection of the virus by real-time RT-PCR in throat swab samples from the patients. No patient was initially admitted to the Department of Infectious Diseases and Department of Respiration. Only Case 2 showed pneumonia on admission; however, she did not present a fever when she came to our hospital; therefore, she may have infected the virus at home. The other three patients showed normal lung images when they were admitted to our hospital. They developed a fever and other symptoms related to COVID-19 after radiotherapy or surgery in different departments, which might indicate that the hospital acquired SARS-CoV-2 infection. This was also demonstrated by another study conducted in our hospital [3].

Early studies have demonstrated the decrease in peripheral WBCs and lymphocytes during SARS-CoV-2 infection, and we observed the same phenomenon in these patients, except Case 2. Case 2 had an increased count of B lymphocytes in the blood, which may be attributed to her underlying disease, B-CLL. Moreover, in the other three cases, we found a reduction in all lymphocyte subsets, including CD3^+^CD4^+^ helper T cells, CD3^+^CD8^+^ cytolytic T cells, B cells, and NK cells. Peripheral lymphopenia was also observed during another coronavirus, SARS-CoV infection [8, 9]. In our study, we found that the counts of CD3^+^ CD4^+^ helper T cells were much lower in two severe cases (Cases 2 and 4) compared to patients with mild (Case 1) or common (Case 3) types of COVID-19. Recently, several groups including our group demonstrated that the reduction of peripheral lymphocytes, especially CD3^+^, CD4^+^ and CD8^+^ T cells, was positively correlated with severity of illness and in-hospital death in SARS-CoV-2 infection [10–12]. However, the peripheral reduction of these T cells might be the result of infiltration and sequestration of lymphocytes in lungs and other organs. The infiltrated lymphocytes were activated in the lung and secreted a lot of cytokines, which might lead the severity of the disease. In a pathological study of a patient who died due to COVID-19, interstitial mononuclear inflammatory infiltrates, dominated by lymphocytes, were found in both lungs [13]. Moreover, the study found that the counts of peripheral CD4^+^ and CD8^+^ T cells were substantially reduced, while their status was hyperactivated [13]. In consistence, a recent study demonstrated that significant decreases in the counts of T cells, especially CD8^+^ T cells, as well as increases in IL-6, IL-10, IL-2 and IFN-γ levels in the peripheral blood in the severe cases compared to those in the mild cases [14]. Taken together, the counts of peripheral lymphocytes, especially of CD4^+^ and CD8^+^ T cells, may be useful in predicting the severity and clinical outcomes in SARS-CoV-2 infection in both tumorous patients and general population.

The clinical outcomes of COVID-19 are determined by virous parameters such as age, underlying diseases, severity of the pneumonia, and admission to an ICU [3, 6]. In our four cancer patients, Cases 2 and 4 were diagnosed as severe cases of COVID-19, and Cases 1 and 3 were diagnosed as mild and common COVID-19, respectively, according to the diagnostic and treatment guideline for SARS-CoV-2 infection issued by Chinese National Health Committee (5th edition). Case 2 was suffering from uncontrolled B-CLL, along with many underlying diseases including COPD, hypertension, and coronary heart disease. This condition and the greater age might have caused the severity of COVID-19 and might have led to the death of the patient. Results from the laboratory showed an elevation in markers of bacterial infection, procalcitonin, and C-reactive protein, after admission and diagnosis of COVID-19. Though antibiotics were used, the contaminated bacterial infection might also have contributed to the death of this patient, which was indicated by the further increases of procalcitonin, and C-reactive protein (Table 2). Case 4 was diagnosed as having COVID-19 at 10 days after liver transplantation. The immunosuppressive agent tacrolimus, which was used for anti-rejection, might have inhibited the host immune system and might have led to the severity of COVID-19. Consistent with this, we found an extreme reduction in lymphocytes including CD3^+^ T cells, B cells, and NK cells in this patient. However, the patient entered into remission of COVID-19 after comprehensive therapy. Liver transplantation was also attributed to the severity of pneumonia. Cases 1 and 3 had better control of their related tumors and had a low risk of immunosuppression. This might have contributed to the success in recovery from COVID-19 after SARS-CoV-2 infection. The durations of SARS-CoV-2 RNA detection in our three survived patients were 18 days (Case 1), 23 days (Case 3) and 39 days (Case 4) as measured from the first time of positive detection of the virus in throat swab samples (Table 2). A recent study demonstrated that a median duration of SARS-CoV-2 RNA detection was 17 days as measured from illness onset in general population [15]. It seemed that the clearance of SARS-CoV-2 was delayed in our tumor patients, especially Case 4, who received immunosuppressive agent tacrolimus after liver transplantation. Thus, the immunosuppressive state in tumor patients might contribute to the delayed virus clearance. Further large-scale studies are needed to verify this hypothesis.

Recently, two studies from China demonstrated a higher infection rate of SARS-CoV-2 in tumor patients than that in general population [7, 16]. Moreover, it seemed that patients with cancer have more severe COVID-19 symptoms than those without [7]. Receiving the cancer treatment such as chemotherapy or surgery in the past month was associated with severe clinical events among those with cancer [7]. In our study, we also found that the uncontrolled B-CLL comorbidity, liver transplantation and receiving immunosuppressive agent were associated with severity of illnesses in Cases 2 and 4.

This study had some limitations. Firstly, the limited numbers of cancer patients were included in our study. This led to difficulty in conduction of a comparison study between cancer patients and general population. Secondly, due to no definite effective drugs against SARS-CoV-2 during early February 2020, all the patients in this study did not receive specific antiviral treatment. It will be interesting to know whether antiviral treatment against SARS-CoV-2 may influence the severity or outcomes of COVID-19 in cancer patients. Thirdly, we speculated that the immunosuppressive state in cancer patients might lead to severity of COVID-19 after their infection of SARS-CoV-2. However, we did not find a specific marker that could reflect the suppression of immune system in cancer patients. The reduction of peripheral CD3^+^, CD4^+^ and CD8^+^ T cells is a universal phenomenon for all SARS-CoV-2 infected patients, not only in cancer patients.

Despite the limited numbers of cancer patients in our and others’ studies, these data emphasizes that the treatment of SARS-CoV-2 infection in cancer patients is challenged by the immunosuppressive state of patients under chemotherapy or surgery [7]. Further large-scale or multicenter studies are needed to compare the clinical features of COVID-19 disease between cancer patients and the general population, and clarify whether the clearance of the virus and the recovery of COVID-19 are delayed in cancer patients.

## Data Availability

The datasets supporting the conclusions of this article are included within the article. And further detailed datasets for the current study are available from the corresponding author on reasonable request.

## Abbreviations

SARS-CoV-2: Severe acute respiratory syndrome coronavirus 2
COVID-19: Coronavirus disease 2019
CT: Computed tomographic
RT-PCR: Reverse transcription polymerase chain reaction
WBCs: White blood cells
NK: Natural killer
B-CLL: B cell lymphocytic leukemia
COPD: Chronic obstructive pulmonary disease
ICU: Intensive care unit
HCC: Hepatocellular carcinoma

## References

[1] Hui DS, Azhar EI, Madani TA, Ntoumi F, Kock R, Dar O (2020). The continuing 2019-nCoV epidemic threat of novel coronaviruses to global health - the latest 2019 novel coronavirus outbreak in Wuhan, China. Int J Infect Dis.

[2] Jin YH, Cai L, Cheng ZS, Cheng H, Deng T, Fan YP (2020). A rapid advice guideline for the diagnosis and treatment of 2019 novel coronavirus (2019-nCoV) infected pneumonia (standard version). Mil Med Res.

[3] Wang D, Hu B, Hu C, Zhu F, Liu X, Zhang J (2020). Clinical characteristics of 138 hospitalized patients with 2019 novel coronavirus-infected pneumonia in Wuhan, China. JAMA.

[4] World Health Organization (WHO). Coronavirus disease (COVID-2019) situation reports. https://www.who.int/emergencies/diseases/novel-coronavirus-2019/situation-reports Accessed 19 Feb 2020.

[5] Lu H (2020). Drug treatment options for the 2019-new coronavirus (2019-nCoV). Biosci Trends.

[6] Chen N, Zhou M, Dong X, Qu J, Gong F, Han Y (2020). Epidemiological and clinical characteristics of 99 cases of 2019 novel coronavirus pneumonia in Wuhan, China: a descriptive study. Lancet..

[7] Liang W, Guan W, Chen R, Wang W, Li J, Xu K (2020). Cancer patients in SARS-CoV-2 infection: a nationwide analysis in China. Lancet Oncol.

[8] Li T, Qiu Z, Zhang L, Han Y, He W, Liu Z (2004). Significant changes of peripheral T lymphocyte subsets in patients with severe acute respiratory syndrome. J Infect Dis.

[9] He Z, Zhao C, Dong Q, Zhuang H, Song S, Peng G (2005). Effects of severe acute respiratory syndrome (SARS) coronavirus infection on peripheral blood lymphocytes and their subsets. Int J Infect Dis.

[10] Wang F, Nie J, Wang H, Zhao Q, Xiong Y, Deng L (2020). Characteristics of peripheral lymphocyte subset alteration in COVID-19 pneumonia. J Infect Dis.

[11] Xu B, Fan CY, Wang AL, Zou YL, Yu YH, He C (2020). Suppressed T cell-mediated immunity in patients with COVID-19: a clinical retrospective study in Wuhan. China J Infect.

[12] He R, Lu Z, Zhang L, Fan T, Xiong R, Shen X (2020). The clinical course and its correlated immune status in COVID-19 pneumonia. J Clin Virol.

[13] Xu Z, Shi L, Wang Y, Zhang J, Huang L, Zhang C (2020). Pathological findings of COVID-19 associated with acute respiratory distress syndrome. Lancet Respir Med.

[14] Liu J, Li S, Liu J, Liang B, Wang X, Wang H (2020). Longitudinal characteristics of lymphocyte responses and cytokine profiles in the peripheral blood of SARS-CoV-2 infected patients. EBioMedicine..

[15] Xu K, Chen Y, Yuan J, Yi P, Ding C, Wu W, et al. Factors associated with prolonged viral RNA shedding in patients with COVID-19. Clin Infect Dis. 2020; ciaa351. doi: 10.1093/cid/ciaa351. 10.1093/cid/ciaa351 PMC7184421 32271376

[16] Yu J, Ouyang W, Chua MLK, Xie C. SARS-CoV-2 Transmission in Patients With Cancer at a Tertiary Care Hospital in Wuhan, China. JAMA Oncol. 2020; e200980. doi: 10.1001/jamaoncol.2020.0980. 10.1001/jamaoncol.2020.0980 PMC7097836 32211820

